# Water-Soluble Coenzyme Q10 Inhibits Nuclear Translocation of Apoptosis Inducing Factor and Cell Death Caused by Mitochondrial Complex I Inhibition

**DOI:** 10.3390/ijms150813388

**Published:** 2014-07-31

**Authors:** Haining Li, Guisheng Chen, Wanrui Ma, Ping-An Andy Li

**Affiliations:** 1 Department of Neurology, General Hospital of Ningxia Medical University, Ningxia Key Laboratory of Cerebrocranial Diseases, Incubation Base of National Key Laboratory, Yinchuan 75004, China; E-Mails: lhnwww@126.com (H.L.); cgslqm@sina.com (G.C.); wma@nccu.edu (W.M.); 2 Department of Pharmaceutical Sciences, Biomanufacturing Research Institute Biotechnology Enterprise (BRITE), North Carolina Central University, 1801 Fayetteville Street, Durham, NC 27707, USA

**Keywords:** apoptosis, apoptosis-inducing factor, Coenzyme Q10, mitochondrion, neuron, reactive oxygen species, mitochondrial membrane potential, rotenone, superoxide, ubiqunone, water-soluble Coenzyme Q10

## Abstract

The objectives of the study were to explore the mechanism of rotenone-induced cell damage and to examine the protective effects of water-soluble Coenzyme Q10 (CoQ10) on the toxic effects of rotenone. Murine hippocampal HT22 cells were cultured with mitochondrial complex I inhibitor rotenone. Water-soluble CoQ10 was added to the culture media 3 h prior to the rotenone incubation. Cell viability was determined by alamar blue, reactive oxygen species (ROS) production by dihydroethidine (DHE) and mitochondrial membrane potential by tetramethyl rhodamine methyl ester (TMRM). Cytochrome *c*, caspase-9 and apoptosis-inducing factor (AIF) were measured using Western blotting after 24 h rotenone incubation. Rotenone caused more than 50% of cell death, increased ROS production, AIF nuclear translocation and reduction in mitochondrial membrane potential, but failed to cause mitochondrial cytochrome *c* release and caspase-9 activation. Pretreatment with water-soluble CoQ10 enhanced cell viability, decreased ROS production, maintained mitochondrial membrane potential and prevented AIF nuclear translocation. The results suggest that rotenone activates a mitochondria-initiated, caspase-independent cell death pathway. Water-soluble CoQ10 reduces ROS accumulation, prevents the fall of mitochondrial membrane potential, and inhibits AIF translocation and subsequent cell death.

## 1. Introduction

Parkinson’s disease (PD) is a common neurodegenerative disease in the aging population. PD is characterized by the selective death of dopaminergic neurons in the substantia nigra leading to low levels of dopamine in the striatum [1]. The etiology of PD is still elusive. Oxidative damage and mitochondrial dysfunction have been proposed to play a major role in the pathogenesis of PD [2]. Rotenone, a widely used pesticide, has been used for many years as a tool to induce PD model *in vitro* and *in vivo* [3,4,5] and proven to be reproducible. Rotenone inhibits complex I of the mitochondrial electron transporter chain (ETC). Its neurotoxicity may be related to the ability of generating reactive oxygen species (ROS) and disrupting mitochondrial oxidative phosphorylation. Elevated ROS levels cause depolarization of the mitochondrial membrane and release of pro-apoptotic factors such as apoptosis inducing factor (AIF), which eventually causes neuronal death [6].

Coenzyme Q10 (CoQ10), also known as ubiquinone Q10, is an electron transporter that transports electrons from ETC complex I and complex II to complex III. In addition, CoQ10 also possesses antioxidant and anti-apoptotic effects [7,8,9]. CoQ10 has lipid-soluble and water-soluble formulas. Most CoQ10 from commercial sources is lipid-soluble and has low bioavailability. In recent years, water-soluble CoQ10 has been developed. Compared with lipid-soluble CoQ10, the water-soluble formula has a much better bioavailability when administered orally [10]. Rotenone has been shown to activate a mitochondria-initiated, caspase-dependent cell death pathway. It is controversial whether rotenone can also activate a caspase-independent cell death pathway. In this study, we first investigated the effects of water-soluble CoQ10 on rotenone-induced neuronal cell death. We then measured ROS production, mitochondrial membrane potential and AIF nuclear translocation. We finally detected cytochrome *c* release and caspase-9 activation.

## 2. Results and Discussion

### 2.1. Effects of Rotenone on Viability of HT22 Cells

**Figure 1:**
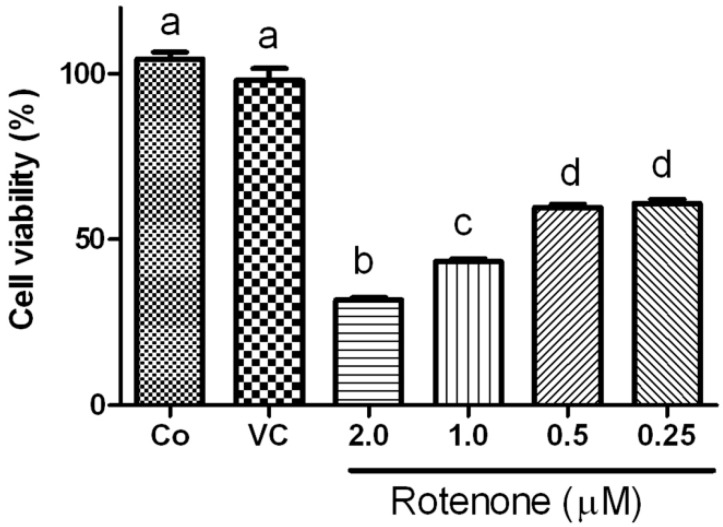
Cell viability after rotenone exposure assayed by alamar blue. Rotenone, at concentrations of 2.0, 1.0, 0.5 or 0.25 µM, induced significant reduction of cell viability compared to control. Data were collected from 3 independent experiments and presented as means ± s.d. Groups with same letter labeled on the top indicate no significant difference while groups with different letter suggest a statistical significance when compared among the groups (*p* < 0.01, b, c, d *vs.* a).

To define the adequate dosage of rotenone for inducing a targeted ~50% cell death, HT22 cells were incubated with four different concentrations (0.25, 0.5, 1.0, 2.0 µM) of rotenone for 24 h. As shown in Figure 1, cell viability decreased with increasing concentrations of rotenone. Naïve (Co) and vehicle (VC, 0.1% DMSO) treated control cells had a viability close to 100%. Rotenone at 0.25 and 0.5 µM resulted in 40% cell death, while rotenone at 2 µM caused 70% cell death. Viability was about 50% in the 1.0 µM rotenone group. As a result, this 1.0 µM rotenone was selected for subsequence experiments.

### 2.2. Protective Effects of CoQ10 against Rotenone Toxicity

**Figure 2:**
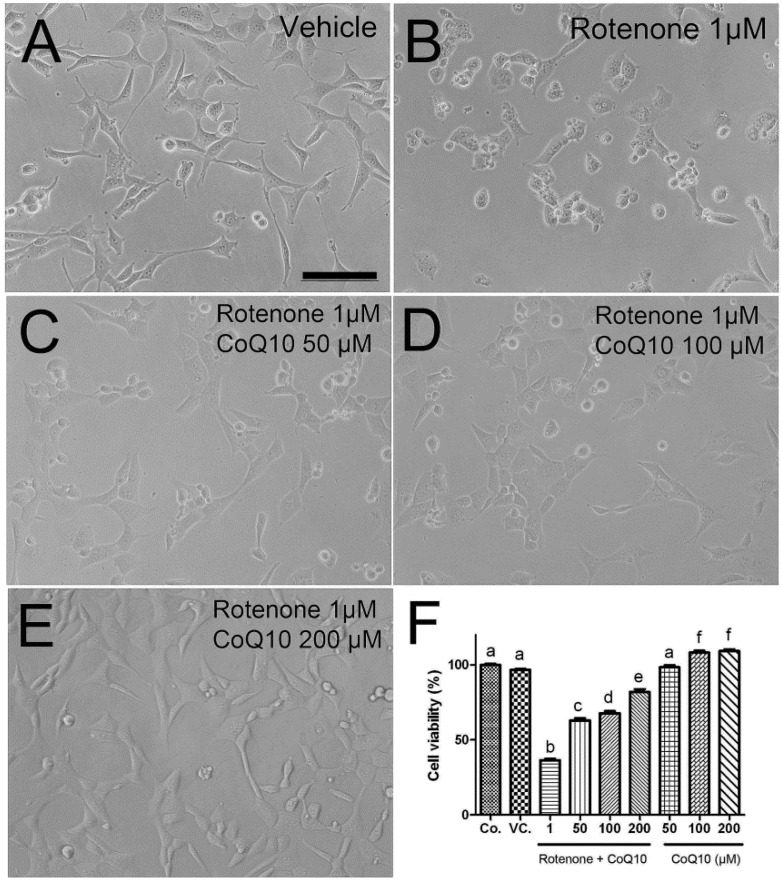
Cell viability in rotenone and CoQ10 incubated HT22 cells. (**A**–**E**) representative photomicrograms showing cell morphology after 24 of rotenone and/or Co-Q10 treatments; (**F**) a summarizing bar graph showing cell viabilities in naïve control (Co), vehicle control (VC), rotenone (1 µM) + Co-Q10 (50–200 µM) and Co-Q10 (50–200 µM) without rotenone groups. Data were collected from 3 independent experiments and presented as means ± s.d. Groups with same letter labeled on the top indicate no significant difference while groups with different letter suggest a statistical significance when compared among the groups (*p* < 0.01). Bar = 200 µm.

To determine the efficacy of water-soluble CoQ10 on rotenone-induced cell death, three dosages (50, 100, 200 µM) of CoQ10 were tested. The cells were treated with CoQ10 for 3 h prior to 1 µM rotenone exposure. Cell viability was assessed by alamar blue at 24 h after rotenone addition. As shown in Figure 2, water-soluble CoQ10 itself did not induce stress to the cell as the viability was close to 100% when cells were treated with CoQ10 alone with dosages of 50, 100, and 200 µM. The efficacy of CoQ10 against rotenone toxicity is in a dose-dependent manner. At the concentration of 50 µM, CoQ10 significantly increased the percentage of viable cells from 40% in 1.0 µM rotenone incubated cells to 60% in CoQ10 treated cells. When the CoQ10 concentration increased to 200 µM, the percent of viable cells further increased to 80% (Figure 2). Because 200 µM CoQ10 displayed the best efficacy, this concentration was chosen for subsequent CoQ10 experiments.

### 2.3. Effects of CoQ10 on ROS Production

**Figure 3:**
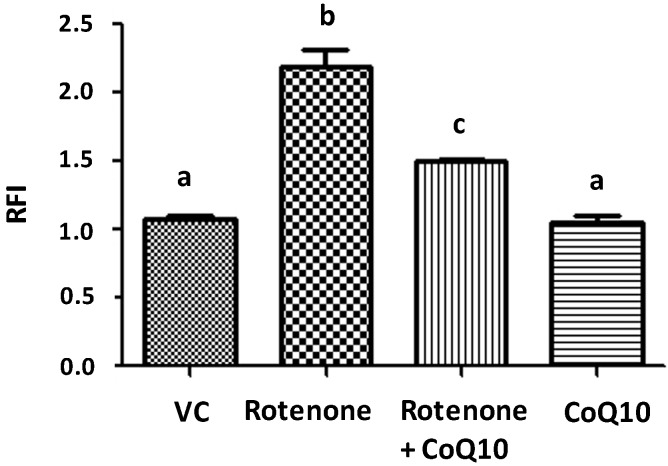
Measurement of ROS level by DHE. Rotenone increased while CoQ10 decreased ROS levels. Data were collected from 3 independent experiments and presented as means ± s.d. Groups with same letter labeled on the top indicate no significant difference while groups with different letter suggest a statistical significance when compared among the groups (*p* < 0.01 b *vs.* a and *p* < 0.05 c *vs.* b).

DHE is oxidized by superoxide to form ethidium and precipitated on the sites where superoxide anions were produced. Rotenone incubation for 24 h induced a more than 100% increase in the production of superoxide comparing to DMSO vehicle-incubated cells (Figure 3). CoQ10 200 µM pretreatment reduced the superoxide level back to normal. CoQ10 itself did not increase superoxide formation.

### 2.4. Effects of CoQ10 on Mitochondrial Membrane Potential

**Figure 4:**
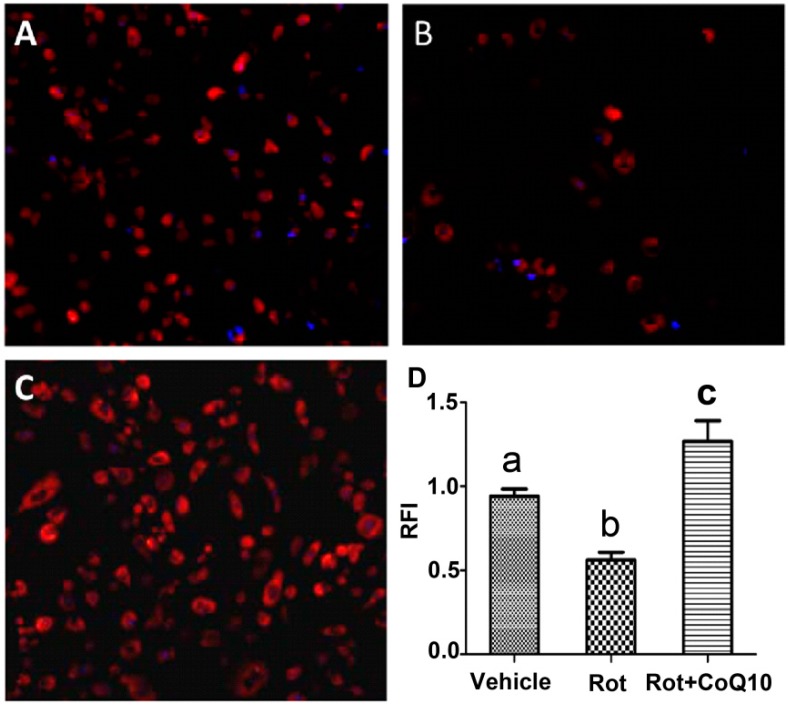
Mitochondrial membrane potential measured by TMRM. (**A**–**C**) representative images collected from (**A**) control, (**B**) rotenone-treated and (**C**) CoQ10 plus rotenone treated cells; (**D**) a summarized bar graph showing depolarization of mitochondrial membrane potential in rotenone-treated cells and preventive effect of CoQ10. Data were collected from 3 independent experiments and presented as means ± s.d. *p* < 0.01 b *vs.* a; *p* <0.05 c *vs.* a. Red color denotes TMRM staining and blue for DAPI.

Mitochondrial membrane potential decreased significantly in cells incubated with rotenone. Pretreatment with 200 µM CoQ10 prevented the depolarization of mitochondrial potential caused by rotenone (Figure 4).

### 2.5. Blockade of Rotenone-Induced AIF Nuclear Translocation by CoQ10

**Figure 5:**
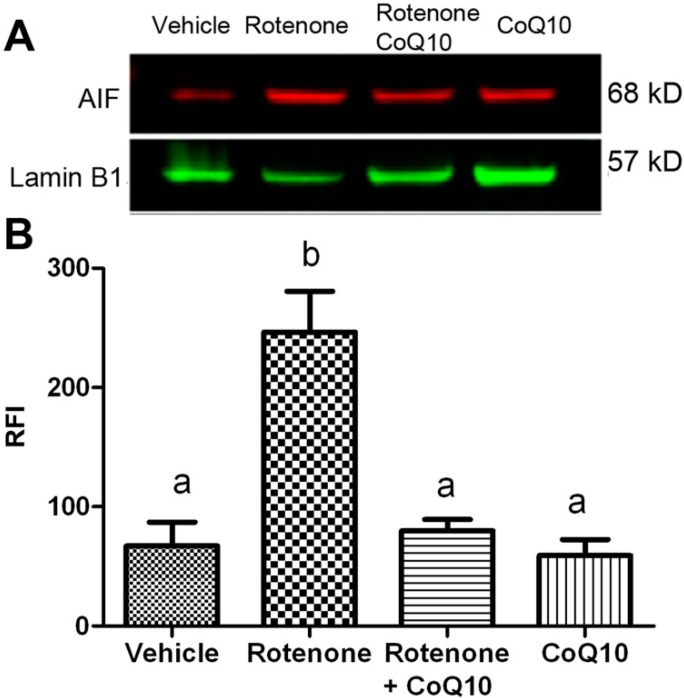
AIF protein levels in the nuclear fractions. (**A**) a representative Western blot showing AIF levels in the nuclear fractions. Lamin B1 served as internal protein loading control; (**B**) a bar graph showing semiquantitative band intensity ratio between AIF and lamin B1. Data were collected from 3 independent experiments and presented as means ± s.d. Groups with same letter labeled on the top indicate no significant difference while groups with different letter suggest a statistical significance when compared among the groups (*p* < 0.01 a *vs.* b).

AIF normally localized in the mitochondria. When cells are under stress or injury conditions, AIF relocated to the nuclei where it causes DNA and nuclear envelope damage. Our results showed that rotenone increased AIF level in the nuclear fraction after 24 h of incubation. Treatment of rotenone-incubated cells with 200 µM water soluble CoQ10 significantly reduced the protein level of AIF in the nuclear fraction (Figure 5), suggesting a blockade of AIF nuclear translocation by the CoQ10.

**Figure 6:**
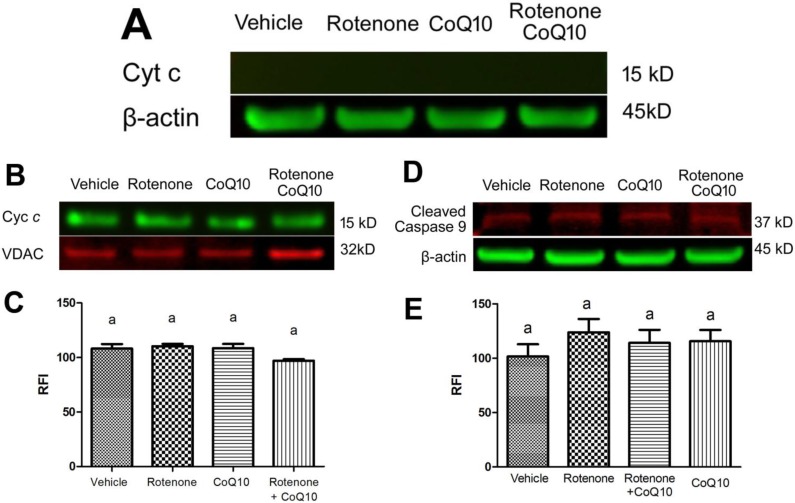
Cytochrome *c* and cleaved caspase-9 levels in the cytosolic and mitochondrial fractions. Equal amounts of protein (20 µg) were loaded into each well. (**A**) cytochrome *c* could be hardly traced in the cytosolic fraction; (**B**) Cytochrome *c* level in the mitochondrial fraction. VDAC served as an internal protein loading control for mitochondrial fraction; (**C**) A bar graph showing semiquantitative band intensity ratio between cytochrome *c* and VDAC. Groups with same letter labeled on the top of each column indicate no significant difference (*p* > 0.05) in the mitochondrial fraction; (**D**) Cleaved caspase-9 in the cytosolic fraction. β-actin served as an internal protein loading control; (**E**) A bar graph showing semiquantitative band intensity ratio between cleaved caspase-9 and β-actin. Groups with same letter labeled on the top of each column indicate no significant difference (*p* > 0.05).

In order to verify whether rotenone also activates a mitochondria-initiated, caspase-dependent cell death pathway, we detected cytochrome *c* in the cytosolic and mitochondrial fractions. As shown in Figure 6A, not even traceable level of cytochrome *c* could be detected in the cytosolic fractions after 24 h rotenone incubation. This was supported by the results showing no decrease of cytochrome *c* in the mitochondrial fraction (Figure 6B,C) and no increase of cleaved caspase-9 in the cytosolic fraction (Figure 6D,E).

### 2.6. Discussion

In this study, we have revealed that rotenone induces neuronal death in a dose-dependent manner and water-soluble CoQ10 exerts a potent protection against rotenone-induced cell death. We have further demonstrated that the neuroprotective effect of CoQ10 is associated with its ability in preventing ROS increase, blocking mitochondrial membrane depolarization and inhibiting AIF translocation from the mitochondria to the nuclei.

In present study, ROS levels increased significantly in rotenone incubated HT22 cells, which is consistent with previous reports [11]. ROS are mainly produced in mitochondrial electron transport chain complexes I and III due to incomplete oxygen reduction. Rotenone binds to complex I, impeding the electron transport from complex I to III leading to the generation of superoxide [12]. It has been proposed that rotenone results in mitochondrial dysfunction and apoptosis through enhancing the production of ROS [13,14].

It is well known that increases in ROS production can trigger the formation of the mitochondrial permeability transition pore (MPTP). The MPTP causes the mitochondria to release proapoptotic proteins including cytochrome *c*, Smac/DIBLO, endonuclease G (EndoG) and AIF [15,16,17,18], which eventually result in cell death. Rotenone chronically intoxicated rats show increased release of cytochrome *c* and activation of caspase-3 [19]. Human Parkinson’s disease brain samples have increased nuclear localization of EndoG [20]. AIF is normally localized in the mitochondrial intermembrane space [21]. Under pathological situations such as oxidative stress, AIF is released from the mitochondria to the nucleus to activate a mitochondria-initiated, caspase-independent cell death pathway, also called AIF-mediated apoptosis. In a previous study, Li and colleagues observed that rotenone induced cytochrome *c* and AIF release; but it did not induce caspase-9 and -3 activation in undifferentiated human neural stem cells [22]. However, cleaved caspase-9 and -3 were increased by rotenone when differentiated human neural stem cells were used [22]. In this study, we have observed that rotenone incubation causes a drastic increase of AIF in the nuclear fraction, suggesting activation of a mitochondria-initiated, caspase-independent cell death pathway. To further examine whether rotenone also activates a caspase-dependent cell death pathway, protein contents of cytochrome *c* and cleaved caspase-9 were measured in the mitochondrial and/or cytosolic fractions 24 h after rotenone incubation. The results showed that a caspase-dependent pathway was not activated since cytochrome *c* was not released into the cytosol and caspase-9 was not activated. These results further strengthened the conclusion that rotenone activates an AIF-mediated cell death pathway. AIF could be leaked out from mitochondria through the MPTP or a Bax-mediated channel on the mitochondrial membrane. Our results showed a significant depolarization of mitochondrial membrane potential in rotenone-incubated cells, suggesting AIF is probably released through the MPTP.

In this study, we have observed rotenone increases both production of ROS and nuclear translocation of AIF and decreases mitochondrial membrane potential in HT22 cells. Such phenomenon has also been observed in SH-SY5Y cells exposed to oxygen-glucose deprivation [23] and in MRC-5 fibroblasts overloaded with calcium [24]. Although it has been reported that ROS can induce mitochondrial AIF release and nuclear translocation, it is not known whether AIF can increase the production of ROS. A recent study shed light on this issue; using an AIF and complex I deficient mouse model, Chinta and colleagues found AIF did not directly modulate the generation of ROS [25]. It is likely that the AIF translocation observed in rotenone-intoxicated cells resulted from high ROS levels.

CoQ10 localizes in the mitochondrial inner membrane and transports electrons from complexes I and II to III. In addition, CoQ10 possesses antioxidant effects [26]. CoQ10 protects cells against ultraviolet B-induced cell death and glutamate cytotoxicity by ameliorating ROS production and inhibiting the release of cytochrome *c* and activation of capase-3 in cultured cells [27,28] and improves neurological functional performance in a rat model of Parkinson’s disease [29]. In present study, we have demonstrated that water-soluble CoQ10 suppresses ROS production, inhibites AIF nuclear translocation, preventes the fall of mitochondrial membrane potential and ameliorates cell death induced by rotenone. CoQ10 may block mitochondrial AIF release through two mechanisms. Firstly, CoQ10 reduces the ROS production, thereby preventing the formation of MPTP. Secondly, CoQ10 may directly bind to the MPTP and inhibit its opening.

## 3. Experimental Section

### 3.1. Chemicals

Dulbecco’s modified Eagle’s medium (DMEM), phosphate buffered saline (PBS), Fetal bovine serum (FBS), Trypsin-Versene Mixture, l-Glutamine and Penicillin-Streptomycin solution were purchased from HyClone laboratories (GE Healthcare Life Sciences, Logan, UT, USA). Water-soluble CoQ10 was purchased from Zymes (Zymes LLC., Hasbrouck Heights, NJ, USA). Rotenone was purchased from Sigma-Aldrich (St. Louis, MO, USA).

### 3.2. Rotenone and CoQ10 Incubation

Murine hippocampal HT22 neuronal cells were cultured in DMEM containing 10% FBS, 2 mM glutamine, and 200 mM streptomycin/penicillin and then maintained at 90%–95% relative humidity in 5% CO_2_ at 37 °C. The culture medium was renewed every 2 days. Cells were seeded in 96-well cell culture plates or T-75cm^2^ flasks and incubated in the above medium for at least 24 h in a CO_2_ incubator to reach 80% confluence. Water-soluble CoQ10 (50, 100, 200 μM) were given 3 h prior to rotenone (0.25, 0.5, 1.0, 2.0 μM) incubation and maintained for 24 h post-rotenone treatment. Cells treated with 0.1% DMSO were served as vehicle controls.

### 3.3. Cell Viability Assay

Alamar blue (Thermo Fisher Scientific, Waltham, MA, USA) was used to determine cell viability. Viable cells take up the dye and the fluorescent intensity could be detected using a plate reader. After treatment, alamar blue reagent (100 µg/mL) was added directly to cell culture medium and incubated for 3 h at 37 °C in a CO_2_ incubator. The plates were read using PHERA Star Microplate Reader (BMG Labtech, Ortenberg, Germany) at the excitation and emission wavelengths of 520 and 570 nm, respectively.

### 3.4. Measurements of Superoxide

Intracellular ROS (superoxide anion) production was measured using dihydroethidine (DHE) fluorescent probe (Life Technologies, Grand Island, NY, USA). Briefly, prior to the termination of each experiment, cells (2 × 10^6^/mL) were incubated with DHE (2.5 μM) for 30 min at 37 °C. The cells were washed, resuspended in PBS and fluorescent intensity was read using a Fluoromax-4 spectrofluorometer (HORIBA Jobin Yvon Inc., Edison, NJ, USA) at the excitation and emission wavelengths of 480 and 590 nm, respectively. Data were presented as relative fluorescence intensity (RFI).

### 3.5. Measurements of Mitochondrial Membrane Potential

Mitochondrial membrane potential was measured using the tetramethyl rhodamine methyl ester (TMRM). TMRM is a cell permeable cationic fluorescent dye that is readily sequestered by active mitochondria. Briefly, HT22 cells were grown on 96-well black/clear plate (BD Biosciences, San Jose, CA, USA) at a density of 5000 cells per well. After treatment with rotenone for 16 h, the cells were incubated with 100 nM TMRM (Life Technologies) and 10 µg/mL Hoechst (Life Technologies) at 37 °C for 30 min. Cells were washed with PBS twice and covered with 200 µL PBS. Stained cells were placed on the stage of Cell INSiGHT NXT High Content Screening Platform (Thermo Fisher Scientific). Images were acquired with a 10× objective (Olympus Tokyo, Japan). TMRM was excited at 549 nm with LED excitation only. Up to 25 fields of view per well were captured. Image processing was performed to quantify the total TMRM fluorescent intensity per cell using Thermo Fisher Scientific HSC studio: Cellomics Scan Version 6.4.4. In brief, TMRM fluorescent intensity was counted in TMRM channel and followed by cell detection using the Hoechst/nuclear channel. The TMRM intensity was tabulated for each cell and then a well-level result was calculated by averaging the TMRM intensity for each cell in a particular well.

### 3.6. Western Blot Analysis

**Figure 7:**
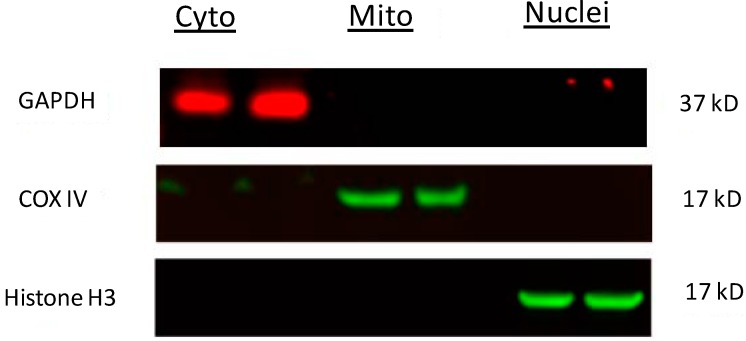
Western blots showing purity of subcellular fractions. Cytosolic, mitochondrial and nuclear fractions were isolated according to the protocol described in the Experimental section and equal amount of proteins (20 µg) were loaded side by side on the same gel. The membrane was probed separately with three primary antibodies against GAPDH, COX IV and Histone H3. A single band for GAPDH at approximately 37 kD in the cytosolic fraction, an obvious band for COX IV at approximately 17 kD in the mitochondrial fraction, and a band for Histone H3 closing to 17 kD in the nuclear fraction was observed. There was no obvious contamination among different fractions.

At 24 h following rotenone and CoQ10 treatments, cells were collected and lysed on ice in a lysis buffer containing 20 mM Tris pH 7.4, 10 mM KCl, 3 mM MgCl_2_, 0.5% NP40 and complete protease inhibitors (EMD Millipore, Billerica, MA, USA). The cell lysates were centrifuged at 500× *g* for 10 min and resulted in a supernatant (S1) and a pellet (P1). The S1 fraction was centrifuged at 20,000× *g* for 20 min and the resulting supernatant was used as a cytosolic fraction. The P1 fraction was washed twice with lysis buffer and resuspended in a lysis buffer containing 1% SDS. The resulting lysates were sonicated briefly with a ultrasonic cell disrupter (Misonix, Farmingdale, NY, USA) and then centrifuged at 20,800× *g* for 30 min. The resulting supernatants were designated as nuclear fractions. The purity of different cellular fractions has been previously demonstrated [30] and also demonstrated in Figure 7. The nuclear fractions were used to detect AIF, mitochondrial and cytosolic fractions for cytochrome *c*, and cytosolic fractions for cleaved caspase-9. Protein contents from each sample were measured using Microplate BCA Protein Assay Kit (Thermo Scientific). Equal amount of protein (20 µg) was loaded into each lane, separated in 4%–12% NuPAGE gels (Invitrogen, Carlsbad, CA, USA), transferred to PVDF membranes (Millipore), and probed with antibodies against: AIF (Cell Signaling, 1:500 dilution), cleaved caspase-9 (Cell Signaling, 1:1000 dilution), cytocrome *c* (Santa Cruz Biotechnology Inc., Santa Cruz, CA, USA), Lamin B_1_ (Life Technologies, 1:1000 dilution), VDAC (Cell Signaling, 1:1000 dilution), and β-Actin (Cell Signaling, 1:1000 dilution), Histone H3 (Cell Signaling, 1:500 dilution), GAPDH (Abcam, 1:10,000 dilution), COX IV (Cell Signaling, 1:800 dilution). Blots were imaged using a LI-COR Biosciences Odyssey Infrared Fluorescent scanner (Lincoln, NE, USA). Both target protein bands and internal loading control protein bands were measured using LI-COR software and presented as a ratio of target protein band fluorescent intensity/loading control protein band intensity.

### 3.7. Statistical Analysis

Data are presented as means ± s.d. One-way ANOVA followed by Newman-Keuls test was used for statistical analysis. A value of *p* < 0.05 was considered statistically significant.

## 4. Conclusions

Rotenone is capable of inducing AIF-mediated cell death. Water-soluble CoQ10 pretreatment protects HT22 cells against rotenone-induced apoptosis via decreasing ROS formation, preventing mitochondrial membrane potential depolarization and inhibiting AIF nuclear translocation.
